# Extracts

**Published:** 1882-12

**Authors:** 


### Extracts.

---

Present Status of Antiseptic Surgery.—Thymol was first brought to notice for this purpose by Volkmann and Ranke, of Halle. At first it promised well, but it has not stood the tests and is now seldom used.

Potassium permanganate, in solution, serves very well for irrigation and washing out purulent cavities; but it is seldom or never employed in the spray. Its solution is excellent, too, for cleansing sponges, or for keeping them

Aluminum acetate was introduced by Maas, who uses $2\frac{1}{2}$ per cent. solution in spray, and soaks his compresses and dressings in the same. He claims that his cases follow a strictly antiseptic course, while the salt certainly is free from irritating qualities. It is deserving of further trial.

Solution of zinc chloride was long ago recommended by Lister for the cleansing and purifying of wounds and sinuses that had become more or less foul. Recently, Kocher has published his experience with the use of weak solutions in the clinic at Berne. He often uses them as weak as one fifth of one per cent., and saturates his dressings with them, thus making it another substitute for carbolic acid. If one may judge from his published statistics, his results are most satisfactory.

Resorcin is a new candidate for favor. The writer has had no personal experience with it, and has hardly seen enough reports of its use to speak intelligently thereof. Like many other new drugs, if all that is said of it be true, it must be of value; but manufacturers' and dealers' statements are not always reliable.

Salicylic acid has the advantage over carbolic acid that neither in strong solution nor in powder has it odor or caustic effect; its disadvantage is its slight solubility in water. It was brought into surgical use by Thiersch, of Leipzig, and has been used a great deal. In the spray it is non-irritating to raw surfaces and serous membranes, and is not infrequently employed.

Volkmann has shown in those cases which I have already referred to where the discharges have a sour odor, and sphæro-organisms are found under the microscope, the use of this acid checks all mischief and odor. Lister makes a cream or ointment of this acid to apply around the edge of other dressings where irritation has been set up. It may be used to advantage in solution for irrigation where carbolic acid is irritating or obnoxious, and dusted dry on ulcerated surfaces, where it dries into the crust and forms a perfect protection from the air.

Alcohol is a most efficient antiseptic; the name of the individual who introduced it having been lost in the dawn of history. As a stimulant injection, more or less dilute, its use is familiar to all. It is desirable here to lay stress on the fact which Hack has demonstrated, that granulations treated by alcohol absorb very little, if at all, while those treated by carbolic acid absorb easily. The practical importance of this fact is very great.

Glycerine has, in the writer's opinion, been too much neglected. Introduced for this purpose by Demarquay, in 1855, it was first tried in hospital gangrene, and then in all wounds. He esteemed it and praised it most highly. I have used it considerably in preserving anatomical specimens and in embalming, and I have usually found that the more severe the test the more satisfactory the result. I had for some time been using carbolized glycerine as a substitute for carbolized oil, it being much more cleanly, and miscible with the water used in washing or irrigating, when I met with articles on the subject by Griffin and others. They advocated its use in major operations, such as severe amputations, etc., claiming that they thus got union by first intention just as when they used the Lister dressing. It has this great advantage that it does not evaporate, and that when mixed with a due proportion of carbolic acid, or alcohol, or iodoform, dressings saturated with it must be either impermeable to the air, or must act as antiseptic filters.

I now thus use it a good deal as a dressing for lesser amputations, lacerated or contused wounds, plastic operations, and the like. I saturate soft canton flannel, or surgeon's lint, or absorbent cotton, with it, apply two to six layers, and then put some gutta percha paper over it, or not, as the case may seem to require, and over all this the usual bandages. With this dressing I have had no bad result, but as complete union by first intention as I ever saw. I can't help venturing the statement, therefore, that the more anatomists and surgeons use glycerin the better they will like it.

Preparations from the eucalyptus, especially the oil, are gradually working themselves into favor.

Boracic acid is too sparingly soluble to be of much service save where it may be applied in dry powder, but in such cases it hardly has a superior. Its crystals are difficult to pulverize, but this difficulty may be overcome by trituration with a few drops of alcohol. It may then be dusted from a pepper box or blown through a blower. Cavities that are to heal by granulation may be filled with it. Thus used after excisions, sequestrotomies, removal of projectiles, etc., it offers the safest means of preventing sepsis. For such purposes its only rival is iodoform, and it has this great advantage, that it is odorless, and that boracic acid posoning is almost unheard of. Lint and cotton may be steeped in hot saturated solution, after cooling and drying the fine crystals are deposited in the meshes of the fibre. Still better is the result if salicylic acid be added. Absorbent cotton thus prepared is equal to the best Lister gauze. Boracic acid is not yet used nearly as much as it ought to be. It has very similar advantages to iodoform without its odor. It certainly stimulates healthy granulations. For my own part, I like it better and better as I use it more and more.

Naphthalin, I imagine, has not yet been tried this side of the ocean. Like boracic acid it is with difficulty reduced to fine powder, but may be used in just the same way and with the same results. Its advantages over everything yet mentioned in this paper is its cheapness. It is a white crystalline body, melting at 79° C., insoluble in water, but easily soluble in four parts of ether.

Lastly, we have iodoform. As an antiseptic it is regarded equal to carbolic acid, and it is perhaps less liable to cause constitutional disturbance. Still, symptoms of poisoning are now and then observed. Iodoform is absorbed in small quantity, and must accordingly be eliminated in some way. This elimination takes place through the kidneys.

The introduction of iodoform has certainly advanced the possibilities of surgery. The great danger formerly after such operations as extirpation of the larynx, thyroid or rectum, excision of the tongue, and the like, was from purulent infection, because from the very nature of the cases a strictly antiseptic dressing was impossible. Now, by the use of this same drug, they are made practicable and even safe. I have seen Billroth stuff a tent of iodoform gauze into the peritoneal cavity after extirpation of the uterus per vaginam, and, leaving it there, tampon the vagina with the same—the patient recovering without a drawback. After operations about the mouth, the wound is stuffed with this gauze, which is changed only after two or three days. After laparotomy, iodoform is sprinkled into the abdominal cavity without hesitation. In a similar manner it is employed after plastic and osteoplastic operations. The various opportunities for its use in syphilitic and skin diseases can only be hinted at here.

In spite of these advantages, it has some bitter enemies, but it is steadily gaining ground. In the writer's humble opinion, it is *the* antiseptic, for surgical purposes, of the immediate future. It will find its largest field, perhaps, in cases of tuberculous joints, sinuses and ulcers. Indeed, its friends claim for it positively and specifically anti-tuberculous effects. This matter should be regarded as still *sub judice.—Roswell Park, M.D., in Chicago Med. Jour. and Ex.*

---

Bacteria.—Time will probably show that many of the pretentious statements which have been made concerning the fungous germ hypothesis of contagious diseases are groundless.

There is, probably, not a part of the body of any one of us of a quarter of an inch in diameter where bacteria-germs are not present. Certainly every time we eat, myriads are carried into our alimentary canal; and every time we breathe, except in the very purest atmosphere, multitudes pass into the air-passages. So small are these bacterial germs, that they would pass without the slightest difficulty through basement membrane and through the interstices of any of the tissues of the organism; and yet the public is taught that there is some intimate connection between bacteria and dust, and morbid phenomena. Erroneous notions are spread far and wide by sensation lectures, under such a title as "Dust and Disease." The dust which causes disease is of a most exceptional kind. It has been said that the air of the Swiss mountains is devoid of bacteria. But is the health and vigor of the inhabitants of the Alps to be compared with that of the workers on the Paddington Dust Heaps? As a fact, ordinary bacteria are harmless enough; they exist in us without disturbing us in any way, but they only grow and multiply in great numbers when circumstances become favorable. I can give you positive proof that bacteria germs exist not only upon the surface of the skin and mucous membranes, but in the internal organs, in the interstices of healthy tissues, and in the blood itself. Some years ago I examined the layers of a fibrinous clot which had been slowly formed from the blood in the interior of a large aneurism of the aorta of a man who died of the disease. The body was examined six or eight hours after death. The aneurism had existed for many years; and probably some of the layers of fibrin which had been deposited were almost as old as the aneurism itself. Now, I found that in all parts of the firm, laminated, leather-like material, which served to greatly increase the wall of the aneurismal sac, there were indications of disintegrating changes having taken place. Upon carefully examining minute pieces of the fibrin under high powers, multitudes of bacteria and their germs were discovered without difficulty. But the older layers in the outer part were here and there softened, and portions of the fibrinous matter seemed eroded, many small masses of soft and broken-down material being present. All these teemed with bacteria, moving, growing and multiplying.

Now, these bacteria, like the fibrin in which they were growing and multiplying, were close to the blood, and within the vascular system; internal to the various tissues constituting the wall of the vessel, which was dilated to form the aneurismal sac. The bacteria must have been growing and multiplying in the lifetime of the patient, and probably for many months before his death occurred. They could not have got into the position in which they were discovered from the outside, for it is hardly conceivable that such an organism as a bacterium could have found out, while outside the body, that within the vascular system there was material suitable for its growth and multiplication. Neither is it possible that the bacteria could have made their way from without to the situation in which they were found, nor could they have effected, in the course of a few hours, the extensive erosions and softening discovered. Such theories could not be sustained with any show of reason. The only conclusion, therefore, which is in accordance with the facts of the case and with common sense, is that which I have before adverted to, viz., that bacteria-germs exist at all times in all parts of the body, even in the blood itself during the healthy state

I conclude that as long as the normal state of things exists, the living bacteria germs in all parts of the organism do not grow and multiply, but when any change occurs of the character of that which results in chemical decomposition, these bacteria-germs multiply. This multiplication proceeds, although we are alive, just as it takes place in dead animal and vegetable matter. And it will occur in every part of every one of us a very few hours after death.

So that you see if bacteria-germs constitute the actual, material, living particles by which contagious disease is propagated, they must be peculiar bacteria, totally different from the ordinary bacteria germs which exist and have existed everywhere. The ordinary bacteria may certainly grow and multiply enormously on the mucous membranes of the body, in follicles of the mucous surfaces and in viscera—intestinal canal, bladder, and passages therefrom, nay, even amongst the elements of healthy growing tissue, without causing any disease at all.

Bacteria-germs, low fungi and low algæ exist in connection with the tissues and fluids of every human organism, and as you may convince yourselves at any time, millions of these are unquestionably present during every moment of existence in health on the dorsum of the tongue. Multitudes, as I have said, pass down the alimentary canal every time we swallow food or fluid. Such ordinary bacteria and their germs do us no harm whatever. But please do not infer from what I have said that putrid fluids loaded with bacteria are innocuous or to be recommended. Organic matter, in a state of putrefactive decomposition, when introduced into the alimentary canal, gives rise to pathological phenomena irrespective of the bacteria it may contain.—*L. S. Beale, M.B., F.R.S., in Slight Ailments.*

---

Fashion in Medicine.—I now come to the second illustration of the effects of fashion in the treatment of disease, namely, the administration of alcoholic stimulants.

Let us see how the matter stands now.

If we carefully consider what has happened, within recent years, as to the administration of stimulants in disease, it is calculated to shake our confidence in the capacity of our profession, as a body, to come to any scientifically based and generally acted upon resolution, as to the proper use of the drug, alcohol, in spite of all the advantages in the medical education of the present day.

What can be thought of the opinion of the profession on this point, or in what estimation it is likely to be held by any one who has paid attention to the subject, when we find such violent fluctuations of opinion as have occurred during the past half century. First, that alcoholic stimulants were withheld in all fevers and inflammatory disorders; next, that strong wines and ardent spirits were given, often in enormous quantities, even to children, in all such cases, as well as in cases of extreme debility; whilst quite recently the medical compass has been boxed again, as we see, by the signatures appended to a document, on the part of a large number of leading practitioners in both London and the provinces, in favor of avoiding, as much as possible, the administration of alcohol in disease, especially to women.

Now, what *are* we to think on the question?

I ask my hearers, does it not show we are at sea on the matter, that we have no principle of any value to guide us, or that we are afraid to formulate it?

Or, are we in this business, as in the old days on the blood-letting question, to manifest our gregarious nature, and all jump through the gap, after the one who jumped first, like so many sheep?

Is any attempt made, in a truly scientific manner, at any medical school in the Kingdom to teach students what the action of alcohol is when given to a sick person, in what form it ought to be administered, and what its dietetic value is—first, in acute disease, and secondly, in chronic, or in the period of convalescence from either? I am afraid not; at least, I do not recollect an instance.

And if so, how are the young practitioners to obtain the information which the authorities of the various schools have neglected to teach?

Then again, why, may I ask, is it not the universal practice when ordering this drug to prescribe the exact dose and frequency of administration, as with other powerful remedies? Surely some distinct directions should be given for gradually diminishing the quantity and frequency of the dose, so as to prevent the formation of intemperate habits. A charge of neglect in this matter is not very infrequently made against the profession as an excuse for the commencement of the habit, though not always with justice.

I am aware that this very important question is, more particularly of late years, complicated by the fact that many persons feel a strong and conscientious objection to the use of alcoholic stimulants at all, and under any circumstances. I am not, however addressing myself to the consideration of individual peculiarities of thought or feeling, for or against, the daily use of some one of the many forms of alcohol.

We all come into contact with the fanatical advocates of total abstinence in every form and under all circumstances, who would exclude it as completely from the sick-room and hospital as from the table. In the face of all this, it behooves us to agree on some definite principle that shall guide us in the use of alcoholic stimulants, and enable us to meet the arguments of the total abstainers. Failing some such action on our part, their societies, ever increasing in numbers and zeal, will assuredly influence general lay opinion in the direction just indicated.

Already pressure has been brought to bear on the managers of many workhouse hospitals on the score of economy, in spite of the individual protests of the responsible medical officers. If not arrested by the authoritative voice of the great majority of the profession, where is this interference with the judgment and freedom of action of medical men to stop?

Ought we, as a trained and educated body of men, with all the evidence before us for coming to a correct conclusion, and specially fitted to deal with questions of treatment on which opinion is likely to be divided, ought we, I say, to allow such questions as I have, however imperfectly, brought to your notice to drift about unsettled until they are taken up by others outside our pale, and decided, as one at least is being decided, with or without our consent? I feel sure that your answer will be in the negative. And if we act according to the best of our ability with singlemindedness, and a regard for truth and the public weal, so shall we secure increased respect for our profession, and so shall we prosper in our work.—*T. Shadford Walker, M.R.C.S., in Med. Press and Circular.*

---

Treatment of Epilepsy.—As these lessons are meant to be essentially practical, we will confine ourselves to the consideration of those methods which appear to-day to give the best results.

Jaccoud very judiciously recommends careful research regarding the causes which led to the development of the malady, so as to obtain thus the principal indications for treatment. Unfortunately these causal indications are often wanting, or exceedingly obscure.

Indications for treatment can often be drawn from the general condition and habits of the patient.

Anæmia and scrofula, for instance, which are so often found associated with epilepsy, should be energetically combatted by remedies appropriate to these morbid conditions; if masturbation is practiced, or the patient constantly commits alcoholic or venereal excesses, every effort should be made to eradicate these bad habits.

Unhappily, however, the causal indications are wanting in most cases, and when they are recognized, it often happens that rational treatment is of no benefit, epilepsy being of such long date that it has become emancipated from its primary cause. In such cases it will be necessary to have recourse to the treatment which, up to the present, has given the best results.

As a general rule the bromides give the best results, and among the bromides, the potash salt seems to be most effectual.

Its efficacy in many cases is incontestable. "The bromide of potash, says Gubler, sometimes cures, often ameliorates, and hardly ever proves of injury, in this disease."

For myself I have experimented with all the new medication lauded in the treatment of the convulsive neuroses, but I return always, in the end, to the bromic salts, which are, in reality, of great efficacy in epilepsy.

The bromides, and particularly the bromide of potash, still forms, then, the basis of treatment; but we must call special attention to the manner of administering them. They should always be administered in solution, experience having demonstrated that their ingestion in the solid state determines grave lesions of the digestive tube. The salt can be administered in almost any of the liquids in common use, in water, wine, milk or bouillion. I give it preferably in one of the aromatic or bitter infusions, with the addition of a small proportion of syrup of orange-peel; these substances slightly stimulate the stomach and aid in the absorption of the drug.

Bromide of potash should be given in doses of one and one half or two drachms daily, but in certain cases of exceptional gravity, either on account of excessive frequency of the attacks or the delirium which follows them, it is found necessary to largely increase the dose, and carry it as high as two and one-half, or even three drachms.

I order it always to be taken just before meals; it is thus better tolerated by the stomach, and the treatment can be longer continued without inconvenience. By administering the bromides methodically, the patient can be maintained constantly under the influence of the drug, each dose taken increasing the action of the preceding dose.

When the attacks have completely disappeared, it will, nevertheless, be necessary to keep the system for months, or even years, under the influence of the drug. This may be effected without the constant administration of the medicament, by giving it at sufficiently near intervals. This is the sole means of rendering the cure complete when it is possible, and of keeping the attacks within bounds when it is impossible to completely eradicate the disease.

The treatment must be continued for several years; but as soon as the attacks have become less frequent and less violent it will be well to suspend treatment for five or six days and then recommence, and continue giving the drug for fifteen or twenty days, and again allow another period of repose. I have remarked that by pursuing this course, the diverse functions, momentarily troubled by the action of the bromides, become reestablished more rapidly than if only one or two days of repose were allowed.

As you have observed in my hospital service, with the patients under constant surveillance, I am in the habit of giving much larger doses of the bromides than could be administered with safety in private practice, for under such conditions it is possible to speedily arrest any accidents due to bromism, if such supervene. The administration of large doses gives rapidly whatever good effects can be obtained from the medicament. Nevertheless, the dose should, as far as possible, be in proper proportion with the profession of the individual, and it should not be forgotten that those who lead a sedentary life have less tolerance for the medicament, and are most subject to the development of toxic symptoms.

In cases where bromide of potash does not prove successful, or where it is not tolerated by the stomach, Charcot and Brown-Sequard have for many years recommended the employment of the other bromides, the bromides of sodium or of ammonium, either singly or associated together, in doses of from 1½ to 2 drachms. I have myself long experimented with the bromide of zinc, and in certain cases it proved successful.

In some cases, no matter what may be the mode of administration, the bromides have no beneficial effect; it will then be necessary to fall back on other methods of treatment.

If the attacks are very mild, coming under the category of those cases to which the term "petit mal" has been applied, or if they consist simply in unconsciousness, lasting but a few seconds, Gubler recommends the extract of belladonna, already employed by Trosseau in similar cases, in doses of one-half to one grain per diem, or sulphate of atropia, in doses of one-fiftieth to one-tenth of a grain per diem.

You are aware, on the other hand, that Herpin has recommended very highly the zinc salts, which gave him good results in certain cases; we have recourse to them at present in cases where the bromides prove inefficacious. I myself employ the oxide of zinc, in doses of from five grains to forty-five grains per diem; the valerianate of zinc, in doses of two to fifteen grains; the lactate, from two to thirty grains, associated either with extract of valerian or conserve of roses.

Herpin also recommended the copper salts, but Mr. Bourneville, who has experimented with them methodically, has never observed any satisfactory results from their administration.

Finally, there is no need to add that these diverse forms of medication may be combined in several ways. One will often be surprised to observe cases which have not been benefited by any one of these medicaments taken singly, much ameliorated when two or more are simultaneously given.

The use of the bromides is followed, in the majority of cases, by a favorable result, even from the debut of treatment. But, after five or six months, it will be often found, to the surprise of the observer, that notwithstanding the continued use of the medicament, the attacks become again as frequent as before the commencement of treatment. It is at this time that a course of bathing and hydro-therapeutic applications will restore to the bromic salt its original efficacy, not only maintaining the amelioration obtained from it, but also rendering it more complete.

It is often necessary to administer tonics in connection with these medicaments, such as the soft extract of quinquina (Fr. cod.), the tincture of bark and cod-liver oil etc. The patients should use only ailments easy of digestion, and the evening meal should be light. This last recommendation is particularly applicable to patients who generally have their attacks at night.

Finally these patients, subject at any moment to lose consciousness, and always under the imminence of an attack, should receive special attention from those about them. They should be cautioned against mounting on chairs or ladders, against remaining too near collections of water or too near the fire; in a word, they should avoid all positions or occupations where a sudden loss of consciousness might prove dangerous. Very many accidents might be avoided by proper attention to such advice.—*M. Magnan, Paris, in Med. and Surg. Reporter.*

---

Writers' Cramp.—Although counting among the minor nervous disorders, the spasmodic disease termed writers' cramp, has probably given rise to quite as much discomfort, and even hardship, as some of those considered more important. For the literary man and copying clerk writers' cramp may mean something not unlike starvation. Many expedients have been tried to relieve it, such as a thick penholder, metal plates attached to the penholder, besides severer measures, such as myotomy, tenotomy and nerve-stretching. The treatment as at present recommended for it consists firstly and fundamentally in the absolute cessation from all attempts at writing for a prolonged period; secondly, in the use of the constant current and a well directed exercise of the muscles with general tonic treatment. The energetic surgeon, Professor Nussbaum, of Munich, comes forward with a method of treatment which certainly deserves trial, if only on account of its simplicity. Considering that, whatever the site of the malady, there is always a spastic contraction of the flexors and adductors with a weak condition of the extensors and abductors, Professor Nussbaum set himself to contrive a penholder that should be directed by the extensors and abductors, instead of the flexors and adductors. This he believes he has accomplished in what he terms a bracelet. This bracelet is a stiff band of gutta percha, oval in shape, about $\frac{1}{8}$ inch thick and $1\frac{1}{4}$ inch broad, having a long diameter of $3\frac{3}{4}$ inches, and a short of $1\frac{1}{4}$ inch. It is therefore wide enough for all five fingers to be slipped into it, but in using it, the thumb is only just entered, the fourth finger is entered almost as far as it will go, and the little finger is left outside. It is evident that the bracelet can he held firmly only by expanding the fingers strongly, that is, by the use of the extensors of the first four fingers, and the abductor of the thumb. To this bracelet the pen is screwed so as to be in contact with the paper when the hand lies on the table. The instrument is made in different sizes by Stiefenholer, of Munich. In order to collect a large experience, Professor Nussbaum advertised, in the newspapers, the gratis treatment of writers' cramp, and had accordingly a considerable number of well marked cases. He states most absolutely that every one of these cases at once wrote easily and distinctly with this instrument, not a trace of spasm appearing in any one of them. All expressed themselves as feeling specially comfortable in using it, and some of the patients, after a time, acquired the feeling that they could again write in the ordinary way without fear of spasm. Professor Nussbaum commends the treatment, therefore, in his preliminary notice of it for trial by those who have such cases under their care. As we have said, it is extremely simple, and has this very strong recommendation in its favor, that in place of the patient being forbidden to write, he is encouraged to write with the instrument as much as he possibly can, in order thereby to strengthen the antagonists of the muscles liable to the spasmodic contraction.—*British Med. Journal.*

---

The Salts of Salicylic Acid in Acute Rheumatism.—The position which salicylic acid is destined to hold among the remedies for acute rheumatism is not yet fully determined. Notwithstanding the strong evidence in its favor which general experience affords, there are not a few able men who hold but a halting faith in its general applicability; and there is important authority in support of the warning that its use is not free from serious danger, and demands considerable caution. The solution of the question must be referred to the teachings of experience; and the following paper is an attempt to make a contribution towards this object, from cases which have fallen under my own observation, chiefly, though not entirely, in hospital practice. * * * *

My ordinary dose for adults, in opening the treatment, has been ten grains repeated every two hours. In eleven cases (out of 51) that dose has been exceeded, to 12, 15 or 20 grains, but rarely, if ever, by myself, though I have occasionally, but rarely, found it necessary to raise the dose at a later period of the case, even up to 25 grains every second hour. The dose I have mentioned as preferred by myself, is, however, considerably less than often employed in this country, and is still further below the dose recommended on the continent. Nevertheless, I have been satisfied with my own moderate quantity; and I prefer it the more that, so far as I have observed, once sickness is produced by salicylic acid, it becomes peculiarly difficult to obtain endurance of even small doses of the medicine afterwards. I have thus far presented what information my cases afford me in recommendation of the salicylate treatment of acute rheumatism; I have yet to enquire what they may report on the opposite side of the question, as to the inconveniences with which the use of the medicine may have been attended; and to what extent the cases afford support to the fears which cling to its employmeut in the minds of many practitioners.

It is, of course, a far easier task to advocate the use of a medicine by proving the advantages it confers, than it is to do so by clearing it of suspicions which have attached themselves to it; the latter undertaking requires a far larger basis of evidence than the former, and involves greater risk of fallacy. It is from the depressing action of the salicylate salts upon the heart that the principal danger seems to be apprehended, and so far as my limited reading extends, it is in this country far more than on the continent that this fear is entertained. It is admitted on all hands as an acknowledged fact, that these salts have a direct tendency to depress the heart's action; but on the continent, at least, it seems the opinion that this tendency is only brought into dangerous operation by large doses.—*James Russell, M.D., F.R.C.P., in Birmingham Med. Review.*

---

Dyspeptic Vertigo.—A good many years ago Mr. Bird found that nitro-muriatic acid would aid digestion, and he found that two or three drops of the strong acid would relieve dizziness from a disordered stomach, if given at the beginning of a meal. He published a report of his successes, and then I made a trial of this remedy, but I did not administer it just as he directed, but gave it after meals, and in five-drop doses in five tablespoonfuls of water, taken through a tube in order to preserve the teeth from the action of the acid, and I often gave ten or fifteen grains of pepsin with it, to aid digestion. I can hardly tell you how many cases of dizziness I have seen cured by this administration. One case I remember was that of a man eminent in politics, who came to me fifteen years ago complaining of dizziness, and I gave him this acid for it. In ten days he came back, and told me he was now well. He then discontinued the use of the remedy, and in three or four months he came back and asked me for the same prescription, and when he began using it he was cured again.

This remedy for dizziness is not in very general use, and it is worthy of being employed far more extensively than it is now I believe. I became very much interested in this class of cases after I had heard of Dr. Bird's plan of treatment, and I now like to see them because I feel that I can give them relief.—*Alonzo Clark, M.D., in Med. Gazette.*

---

Comparative Action of the Bromides.—MM. Cheron and Fouques, having experimented at some length with the three well-known bromides (of potassium, sodium and ammonium), have reached the following conclusions These salts act, by virtue of their bromide, as moderators of the reflex centres. The bromide of potassium joins to its sedative action on the nervous centres a depressing action on the muscular system; it is thus a neuro-muscular agent. The bromide of sodium has an action like that of bromide of potassium on the nervous centres, but does not affect the muscular system; it is simply thus a moderator of reflex action. The bromide of ammonium has, by virtue of its bromide, an action on the nervous system similar to that of the other two, while it is also, by virtue of its ammonia, an excitant and diffusible; it is thus at once a moderator of reflex action and a peripheral excitant.

Consequently, when it is desired to influence the reflex powers and the muscular system, preference should be given to the bromide of potassium; if, however, we wish to act only on the reflex centres, the bromide of sodium is indicated; finally, if, leaving the muscular system out of consideration, it is desired to act on the nervous centres, to restrain the circulation and to effect a diminution in blood pressure, the bromide of ammonium will most probably give the required result.—*Glasgow Medical Journal.*—*Cincinnati Lancet and Clinic.*

---

THE *Birmingham Medical Review* says: "We have heard a good deal about the course taken by the New York Medical Society in sanctioning consultations by its members with duly qualified homœopaths, but it seems that in Canada they go a good deal further, for the College of Physicians and Surgeons of Toronto has a homœopathic member of the examining board, and candidates who intend to register and practice as homœopathic practitioners, on giving notice to the register are allowed to be examined by him. This is rather weak-kneed of the Toronto College of Physicians and Surgeons; it might at least confine its examinations to well ascertained scientific facts, and leave all speculative questions alone. If a student who has been well grounded in medical science chooses, after becoming qualified, to join the homœopathic ranks, he does so with his eyes open; but we should take no part in teaching him or encouraging him to learn a system which we believe to be based upon vain hypotheses, and which has a direct tendency to allay the desire for further progress in pathology and diagnosis to which the medicine of to-day owes most of its triumphs."

